# Biogeography of the theileriosis vector, *Rhipicephalus appendiculatus* under current and future climate scenarios of Zimbabwe

**DOI:** 10.1007/s10493-023-00796-1

**Published:** 2023-05-12

**Authors:** Tinotenda M. Nemaungwe, Ellie M. S. P. van Dalen, Emily O. Waniwa, Pious V. Makaya, Gerald Chikowore, Frank Chidawanyika

**Affiliations:** 1 Division of Veterinary Technical Services, Ministry of Lands, Fisheries, Water and Rural Development, Harare, Zimbabwe; 2 Department of Zoology and Entomology, University of the Free State, PO Box 339, Bloemfontein, 9300 South Africa; 3 Centre for Biological Control, Department of Zoology and Entomology, Rhodes University, Grahamstown, 6140 South Africa; 4 International Centre of Insect Physiology and Ecology (icipe), PO Box 30772-00100, Nairobi, Kenya

**Keywords:** Climate change, Microhabitats, Species distribution modeling, Lethal temperature

## Abstract

Climate directly influences the epidemiology of vector-borne diseases at various spatial and temporal scales. Following the recent increased incidences of theileriosis in Zimbabwe, a disease mainly transmitted by *Rhipicephalus appendiculatus*, we determined lethal temperatures for the species and current and possible future distribution using the machine learning algorithm ‘Maxent’. *Rhipicephalus appendiculatus* larvae had an upper lethal temperature (ULT_50_) of about 44 ± 0.5 °C and this was marginally higher for nymphs and adults at 46 ± 0.5 °C. Environmental temperatures recorded in selected zonal tick microhabitats were below the determined lethal limits, indicating the ability of the tick to survive these regions. The resultant model under current climatic conditions showed areas with high suitability indices to the eastern, northeastern and southeastern parts of the country, mainly in Masvingo, Manicaland and Mashonaland Central provinces. Future predictions as determined by 2050 climatic conditions indicate a reduction in suitable habitats with the tick receding to presently cooler high elevation areas such as the eastern Highlands of Zimbabwe and a few isolated pockets in the interior of the country. Lowveld areas show low suitability under current climatic conditions and are expected to remain unsuitable in future. Overall, the study shows that *R. appendiculatus* distribution is constrained by climatic factors and helps identify areas of where occurrence of the species and the disease it transmits is highly likely. This will assist in optimizing disease surveillance and vector management strategies targeted at the species.

## Introduction

Ticks and tick-borne diseases (TBDs) are major causes of livestock mortalities and subsequent economic losses globally (Abbas et al. 2014). Several tick species are disease vectors of veterinary importance and inflicting huge annual costs for management of the ticks and reduction of the burden of the diseases they transmit (Horak et al. 2009; Spickett et al. 2011; Vudriko et al. 2016). Direct and indirect production losses due to TBDs in sub-Saharan Africa are estimated to be in the range of 10–80%, depending on the disease transmitted (Chepkony et al. 2021). For example, in sub-Saharan Africa, theileriosis (East Coast fever) alone accounts for approximately one million cattle deaths annually resulting in approximately USD 300 million economic losses (Shekede et al. 2021). Direct losses emanate from ticks as blood sucking parasites and indirect losses from ticks as disease vectors which results in reduced growth rate, fertility problems, abortions, decline in meat and milk production, reduced value of hides and livestock mortalities.

Given the economic importance of ticks, there is need for constant surveillance to monitor their current and also project future geographic distribution. Knowledge of tick ecology, physiology and geographic distribution will therefore be vital. However, for ectotherms, the distribution is highly dynamic and is heavily influenced by environmental factors, particularly temperature (Chidawanyika et al. 2020; Zannou et al. 2021). Body temperature among these ectotherms depends on ambient environmental conditions as well as behavioral mechanisms for thermoregulation (Chown and Nicholson 2004; Kearney et al. 2009). Hence, arthropod distribution is mainly dictated by thermal margins with a presumptively higher abundance in areas offering most suitable conditions (Peterson et al. 1999; Estrada-Peña et al. 2016).

In recent years, climate change-mediated shifts in the distribution of organisms have become evident. For instance, Longbottom et al. (2020) predicted a decline in tsetse abundance in traditional endemic low-lying areas whereas previously cooler high-altitude areas are expected to provide suitable habitats in Zimbabwe. These predictions are premised on projected temperature rises which are expected to range between 3 and 4 ºC by 2100 (Engelbrecht et al. 2015). Although some arthropods may counter these changes through mechanisms such as phenotypic plasticity and other adaptive evolutionary responses (Nyamukondiwa et al. 2013; Sgro et al. 2016; Mutamiswa et al. 2017), biogeographical changes are expected for several species, which fail to adapt to changing environments (Parmesan et al. 1999; Forsman et al. 2016). Changes in climatic factors may alter arthropod life-history traits—such as their survival, reproduction and fecundity—and impact the encounter of hosts and pathogen-carrying vectors, thereby affecting disease transmission dynamics and ultimately population dynamics (Nguyen et al. 2014). For instance, Pfaffle et al. (2013) reported that temperatures < 7 °C led to continuous tick inactivity which negatively affects their questing behaviour. Such individual responses to both diurnal and seasonal thermal fluctuations mediate population level effects where severe unfavourable conditions can lead to either migration or extinction.

Even though climatic factors such as temperature and humidity play a role in the distribution of animal disease vectors (Tønnesen et al. 2004), host distribution, vegetation cover and control efforts also modulate observed distributions (Estrada-Pena et al. 2008). Absence of ticks in some areas may not be an indicator of non-conducive eco-physiological requirements as host animal movement is key in the dispersal of ticks into novel environments regardless of the prevailing climatic conditions (Barre and Uilenberg 2010; Sungirai et al. 2018). However, the survival and proliferation of the ticks’ free-living stages in these environments is mainly determined by their adaptability to microclimates, availability of suitable hosts and vegetation cover among other key factors (Greenfield 2011). For example, the presence of *Rhipicephalus microplus* both in the southern and northern Lowveld of Zimbabwe was attributed to animal movement during the land redistribution exercise which affected land ownership, methods of cultivation and farm organisation across the country (Mavedzenge et al. 2008).

Species distribution models have often been used to predict suitable habitats or ecological niches and model potential habitats for ticks based on presence data and environmental covariates (Hahn et al. 2016; Sungirai et al. 2018; Pascoe et al. 2019; Namgyal et al. 2021). Elsewhere, these models have been used to optimize the control of animal disease vectors. For example, Dicko et al. (2014) used a Maxent-derived habitat suitability model to delimit target areas for the implementation of an area-wide integrated tsetse control program in Senegal. Thus, predictive models fill in gaps of a known distribution (Estrada-Pena et al. 2016) and address issues of accessibility and costs which prohibit large-scale intensive surveys (Chikowore et al. 2017; Dobson and Randolph 2011) described this as a ‘top-down’ approach which can be implemented rapidly. Furthermore, the availability of spatially explicit species occurrence records and remotely sensed datasets allows species-specific microenvironments to be identified (Ozdenerol et al. 2008). Putting the predicted potential risk areas under surveillance is an effective way of managing the vector and its associated diseases (Hahn et al. 2016).

In Zimbabwe, the distribution of most tick species previously followed altitude-delimited temperature zones with high prevalence on the Highveld (> 600 m above sea level) (Sungirai et al. 2015). Recent studies have shown shifts for several species, with some becoming established beyond their previous geographical ranges (Gambiza and Nyama 2000). For instance, Sungirai et al. (2017) reported shifts in the distribution of *Amblyomma variegatum* and *R. microplus* ticks in Zimbabwe. The former is reported to have expanded its range from the Lowveld to some parts of the Highveld. The latter shifted from occupying only the eastern Highveld to reach as far as the south-eastern Lowveld, northern Highveld and northern Lowveld of the country offering low temperatures and high rainfall suitable for its survival. Studies by Shekede et al. (2021) have also confirmed these shifts by reporting spatial clustering of *R. microplus* in the north and northeastern districts of the country.

The brown ear tick, *Rhipicephalus appendiculatus*, is highly prevalent in the Highveld (> 600 m asl) areas characterized by high rainfall (≥ 650 mm per year), lower temperatures (10–30 ºC) and adequate vegetation cover (Hove et al. 2008; Sungirai et al. 2015). The tick transmits the parasite *Theileria parva*, which causes theileriosis in bovids. Norval et al. (1991) previously reported that *R. appendiculatus* diapause broke the transmission of the parasite in Zimbabwe. However, the Department of Veterinary Services in Zimbabwe reported an increase in the incidence of the disease and related cattle mortalities over the past 4 years. Some of the incidences were reported in traditionally non-endemic areas and cases also changed from being seasonal to all year round. Therefore, questions arise on the distribution of the disease vector, *R. appendiculatus*. Thus, the overall goal of this study was to model the current and future distribution of habitats suitable for *R. appendiculatus*. Specifically, the study aimed to answer the following questions: (1) what is the current distribution of *R. appendiculatus* and how will this be affected by climate change? And (2) which temperatures are lethal for the tick species and how do these influence current and future distributions? The study hypothesized that the distribution of *R. appendiculatus* is shaped by climatic conditions and these will shape the distribution of the species in future.

## Materials and methods

### The study area

**Fig. 1:**
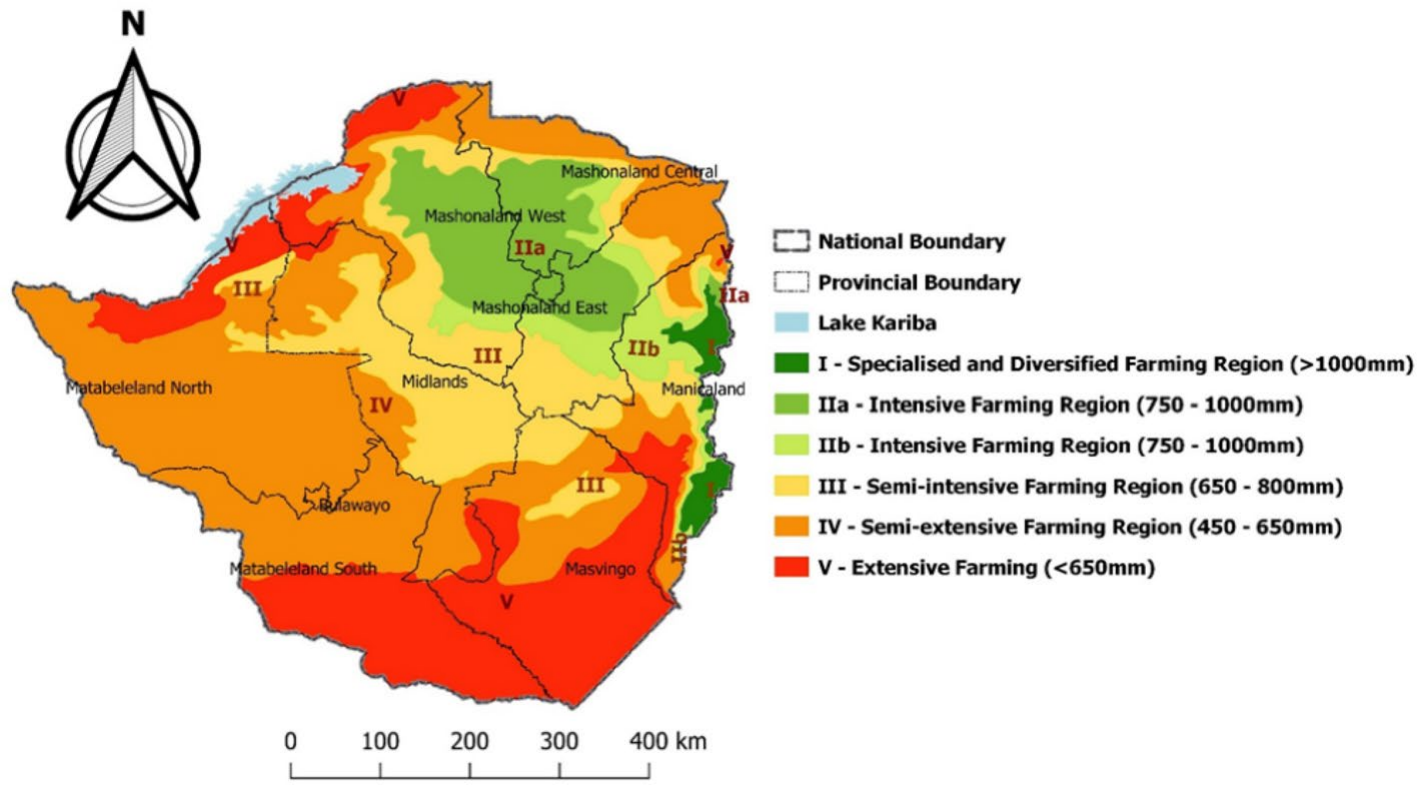
Study area showing the agro-ecological regions of Zimbabwe. (redrawn from Vincent and Thomas 1961)

The study was conducted in Zimbabwe, which lies in the sub-tropical region and is predominantly savanna. The country is divided into five agro-ecological regions based on temperature and rainfall; regions I, II and III lie in the Highveld whereas region V and part of IV are in the Lowveld (Fig. 1; Norval et al. 1994; Sungirai et al. 2018). Rainfall received per annum generally decreases from region I (> 100 mm/year) to V (< 450 mm/year) whereas temperature increases from 10 to 30 °C in the Highveld, to > 30 °C in the Lowveld. Soil type also decreases in quality from heavy textured red clay loams in region I, greyish brown sands and sandy loams derived from granite rocks in regions II and III, to very shallow vertisols in region V. Despite the differences in rainfall regimes, soil types and temperature patterns, agricultural activities are carried out across all regions. Specialized and diversified crop-livestock farming is practiced in region I. Region II and III are intensive and semi-intensive farming regions whereas regions IV and V receive extremely low rainfall (< 500 mm) per annum and are therefore occupied by semi-intensive and intensive beef and game ranching.

### Influence of temperature on survival and distribution of ***Rhipicephalus appendiculatus***

Hygrochron iButton data loggers (DS1923-F5, ± 0.5 °C; Cold Chain Technologies, Franklin, MA, USA) were deployed in tick habitats in selected districts to record microhabitat temperature fluctuations between April and November 2021 at hourly intervals. In Zimbabwe, the highest temperatures are experienced in October whereas June records the lowest temperatures. Hence, the sampling period encompassed months during which temperature extremes are experienced.

To determine the impact of thermal extremes on the survival of *R. appendiculatus*, upper lethal temperatures (ULTs) were assayed using the direct plunge protocol (Chidawanyika et al. 2020; Fieler et al. 2021). Higher temperatures were considered due to heatwaves experienced in the country recently as well as the absence of sub-zero temperatures from environmental data collected during the study. As *R. appendiculatus* is a three-host species, larval, nymphal and adult stages were assayed. For each developmental stage, 10 unfed ticks were placed in five 60-ml screw top polypropylene vials to yield a sample size of n = 50 for each treatment. Lids of the vials were perforated for ventilation and a piece of moistened cotton wool was attached in each vial to maintain relative humidity above 80%. Vials were then placed in Ziploc bags and immersed in a programmable Nuve water bath (Sanayi Malzemeleri, İzmir, Turkey) for 2-h durations at temperatures ranging from 38 to 50 ± 0.5 °C that elicit 0–100% mortality. After the treatment, the vials were incubated in a POL-EKO humidity chamber (POL-EKO Aparatura, Wodzisław Śląski, Poland) at 27 ± 1 °C and 80–90% r.h. for 24 h before scoring survival. Survival was defined as the ability to coordinate normal locomotory response to stimuli such as gentle prodding.

### Tick presence data

Tick presence data was collected during a national tick survey conducted across all agro-ecological zones of Zimbabwe by the Department of Veterinary Services in 2013. This survey was carried out using a stratified sampling approach where agro-ecological zones of Zimbabwe were the strata. Five districts were randomly selected from each stratum except the stratum representing agro-ecological zone I, with three districts which were all sampled. In total, 23 districts were selected and 10 dip tanks, which constituted the sampling units, were sampled from each of the selected districts. At each dip tank, ten animals were randomly sampled (maximum of ten ticks per preferred attachment site). Targeted sites were the head, neck and dewlap, ears, body and belly, legs and tail switch, udder and scrotum. Samples were collected in universal bottles and preserved in 70% alcohol, 5% glycerol and 1% chloroform. Ticks were collected on a monthly basis throughout the year and were identified at the Central Veterinary Laboratory (CVL) using morphological keys by Walker et al. (2003). *Rhipicephalus appendiculatus* presence locations were cleaned in ArcGIS to ensure coordinate precision, remove duplicates and remove wrong coordinates. As a result, 144 presence locations with a spatial distance of 5 km remained and were used to train and evaluate the Maxent model. The distance of 5 km was chosen as it is the catchment area for each dip tank which constituted the sampling unit.

### Environmental covariates

Climatic variables were downloaded from the Worldclim v.2.0 database (Hijmans et al. 2005). As bioclimatic variables are highly correlated and several multicollineality tests have been performed on them, we used results of the hierarchical cluster analysis performed by Mudereri et al. (2021) which produced five clusters based on Pearson’s r as the distance and a correlation coefficient of 0.7 as the cutoff point. From each cluster, variables which best describe the ecology of ixodid ticks were selected to avoid model overfitting and improve the interpretability of the model (Merow et al. 2013). As a result, a total of five bioclimatic variables (Table 1) were used to construct the model.

**Table 1:** Predictor variables used to model suitable habitats for *Rhipicephalus appendiculatus* in Zimbabwe. (source: Worldclim)

Variable	Code
Temperature seasonality	bio4
Maximum temperature of the warmest month	bio5
Temperature annual range	bio7
Precipitation of wettest month	bio13
Precipitation seasonality	bio15

### Data analysis

#### Microclimatic data

To visualize temperature and humidity in tick habitats, readings were plotted against sampling time in Excel (Microsoft Office 2016). Differences in mean temperatures were then compared using one-way ANOVA in STATISTICA v.7 (TIBCO Software, Palo Alto, CA, USA) whereas Tukey-Kramer post hoc tests were used to separate statistically significant groups.

#### Lethal temperature limits

The response of *R. appendiculatus* larvae to increasing temperatures were modelled using R statistical software v.3.3.0 (R core team 2021). The ‘drc’ package (Ritz et al. 2015) was used to construct a 2-parameter log-logistic model with the lower and upper limits fixed at 0 and 1, respectively. The temperature at which 50% of the larvae died was inferred from the model using the ‘ED’ function of the package.

#### Habitat suitability modelling

**Fig. 2:**
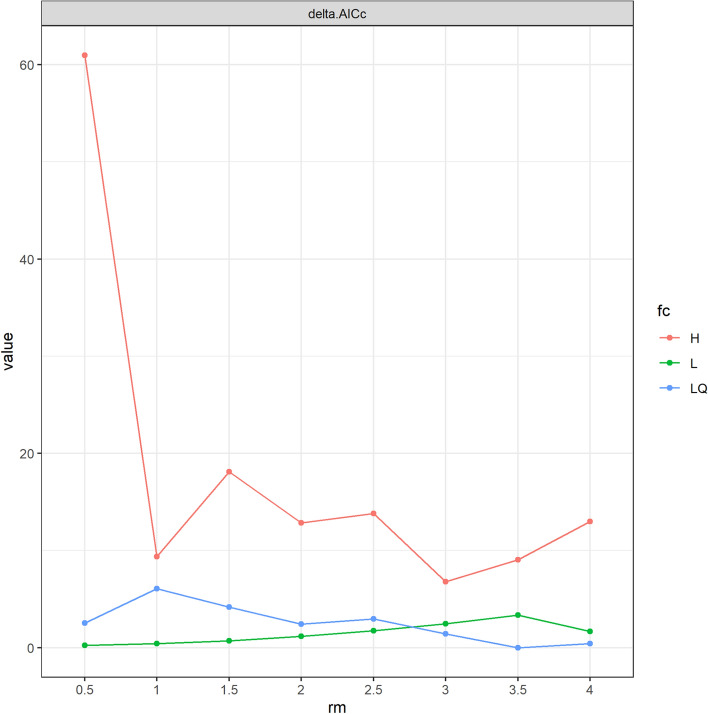
Changes in corrected Akaike Information Criterion (ΔAICc) during model tuning in response to varying feature classes – hinge (H), linear (L), quadratic (Q), product of linear and quadratic (LQ) – and a range of regularization multipliers (rm)

Current and future *R. appendiculatus* habitats were modelled using the maximum entropy technique (Maxent; Phillips et al. 2006). Maxent is a logarithmic technique which uses mathematical principles to determine habitat suitability by comparing conditional density of presence sites with marginal density of an object (Hijmans and Graham 2006). The model uses presence data, randomly selected pseudo-absences as background points and environmental variables to generate a probability distribution across a landscape (Phillips et al. 2006; Elith and Leathwick 2009). Modelling was done using Wallace, an R-based graphic user interface (GUI) application for ecological modeling for building, evaluating, and visualizing models of species niches and distributions that is fully reproducible (Kass et al. 2018). As sampling was stratified covering all the agro-ecological regions, 10,000 background sampling points were selected from the entire country. The presence dataset was randomly split into two folds (*k* = 2) for training and evaluation. Optimum tuning and parameter settings for Maxent with presence-only *R. appendiculatus* observations were derived from the ‘ENMevaluate’ function in the ‘ENMeval’ package within the Wallace GUI. Model parameters with the lowest change in the corrected Akaike information criterion (ΔAICc = 0) were derived from a range of 0.5–4 with an incremental value of 0.5 for linear (L), quadratic (Q), product (P), threshold (T), hinge (H) features (Fig. 2). The model transfer module of the Wallace application was then used to predict future *R. appendiculatus* habitat suitability for the 2050 climate scenario. For this prediction, the Shared Socioeconomic Pathway 2 (SSP2-4.5) was used (Fick and Hijmans 2017; Meinshausen et al. 2020). This pathway predicts that CO_2_ emissions will hover around current levels before beginning to decline by mid-century leading to a 2.7 °C rise by the end of the century. In addition, socio-economic factors are expected to follow their historical trends, coupled with slow progress towards sustainability. Model performance was then assessed quantitatively using the area under the curve (AUC) statistic, derived from threshold-independent receiver operating characteristic (ROC) analysis. The ROC curve is a plot of true positives against false positives with AUC values between 0 and 1. An AUC closer to 1 indicates a high predictive capability of the model. In addition, the Continuous Boyce index (CBI), a measure which only requires presences and measures how much model predictions differ from random distribution of the observed presences across the prediction gradients was used to evaluate model performance (Boyce et al. 2002). Positive CBI values (closer to + 1) indicate a model in which present predictions are consistent with the distribution of presences in the evaluation dataset whereas values closer to 0 mean that the model is not different from a random model.

## Results

### Environmental temperatures in* Rhipicephalus appendiculatus* microhabitats

**Fig. 3:**
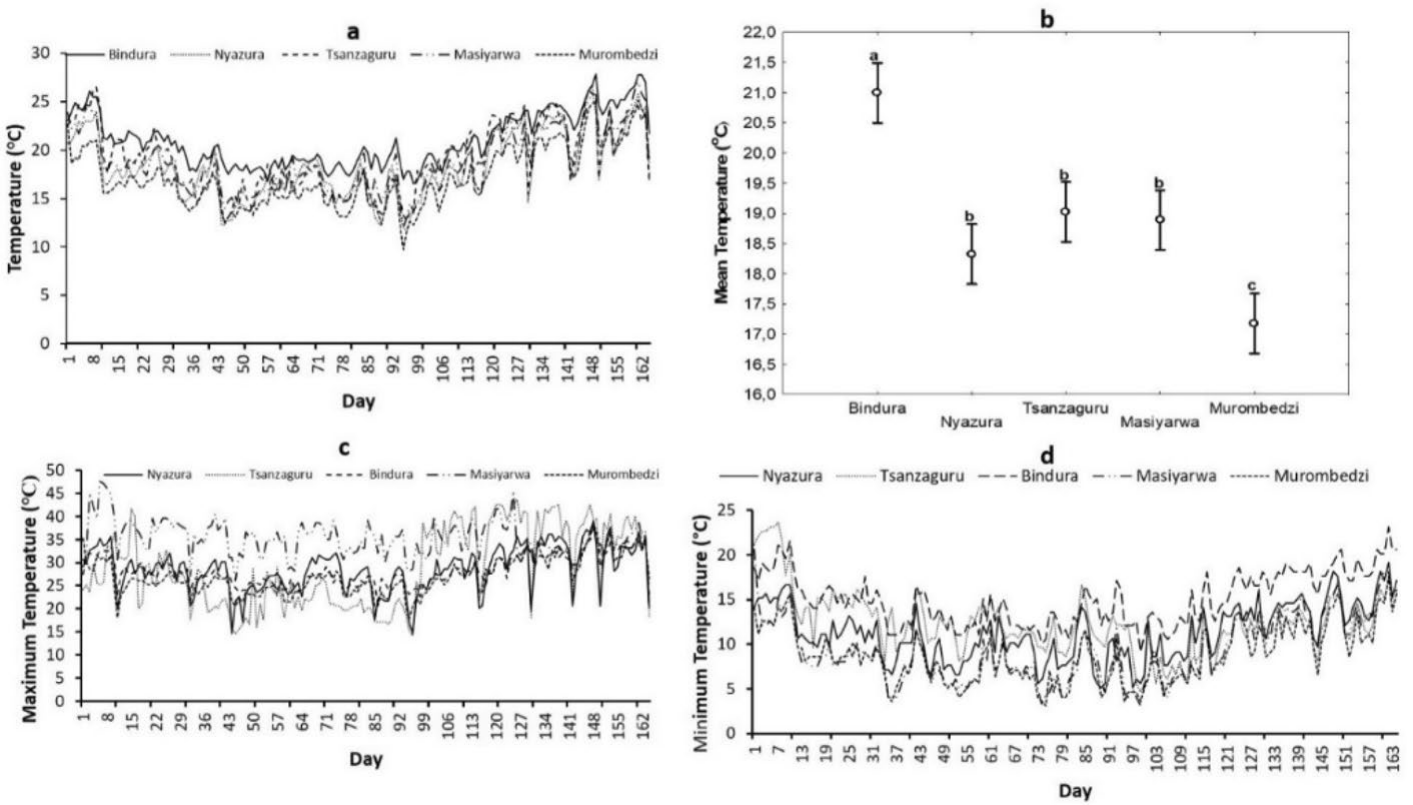
Mean temperature fluctuations (**a**), differences in mean (+ 95% confidence interval) temperatures (**b**), maximum temperature fluctuations (**c**) and minimum temperature fluctuations (**d**) in five selected districts in Zimbabwe. Means in panel **b** capped with different letters are significantly different (Tukey-Kramer test: P < 0.05)

Bindura in Mashonaland Central recorded the highest mean temperatures in tick habitats (Fig. 3a). Post-hoc tests also indicated that mean temperatures were significantly higher at this site, whereas Murombedzi in Mashonaland West had the lowest temperatures (Fig. 3b). There were no significant differences in mean temperatures for Nyazura and Tsanzaguru tick habitats in Manicaland province compared to Masiyarwa in Mashonaland West province. When temperatures were decoupled into minimum and maximum, Masiyarwa in Mashonaland West had the highest maximum temperatures (Fig. 3c) and Murombedzi had the lowest minimum temperatures (Fig. 3d).

#### Upper lethal temperature assays

**Fig. 4:**
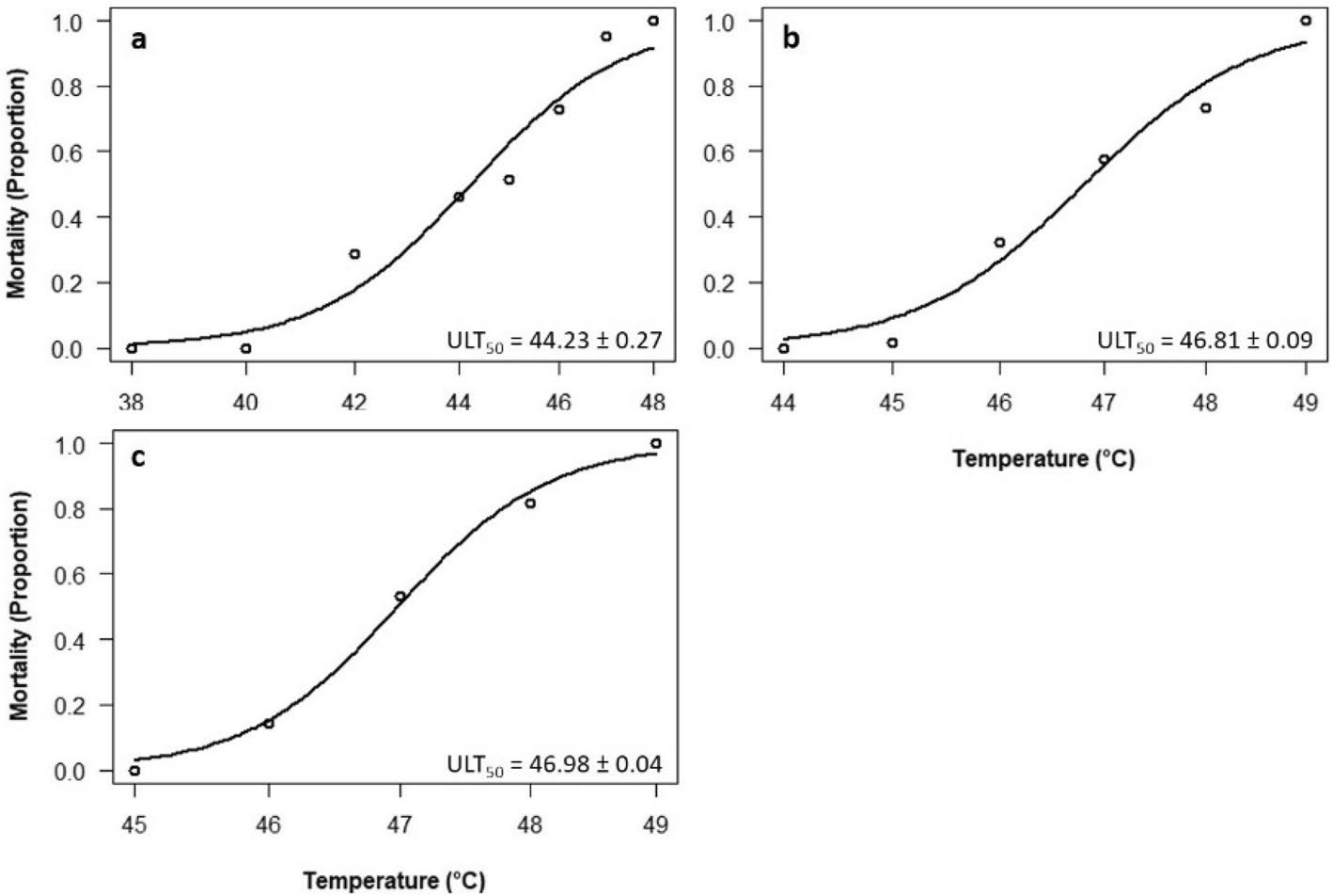
Response of *Rhipicephalus appendiculatus* larvae (**a**), nymphs (**b**) and adults (**c**) to increasing temperature in a 2-parameter log-logistic model (with lower limit at 0 and upper limit at 1) with predicted ULT_50_ ± SE

The 2-parameter log-logistic model predicted the upper lethal temperature (ULT_50_) for *R. appendiculatus* larvae at 44.23 ± 0.27 °C (mean ± SE) whereas those of nymphs and adults were 46.81 ± 0.09 and 46.98 ± 0.04, respectively (Fig. 4). Mortality of *R. appendiculatus* was directly proportional to temperature with an increase in temperature severity resulting in a corresponding increase in tick mortalities.

### Distribution of ***Rhipicephalus appendiculatus***

**Fig. 5:**
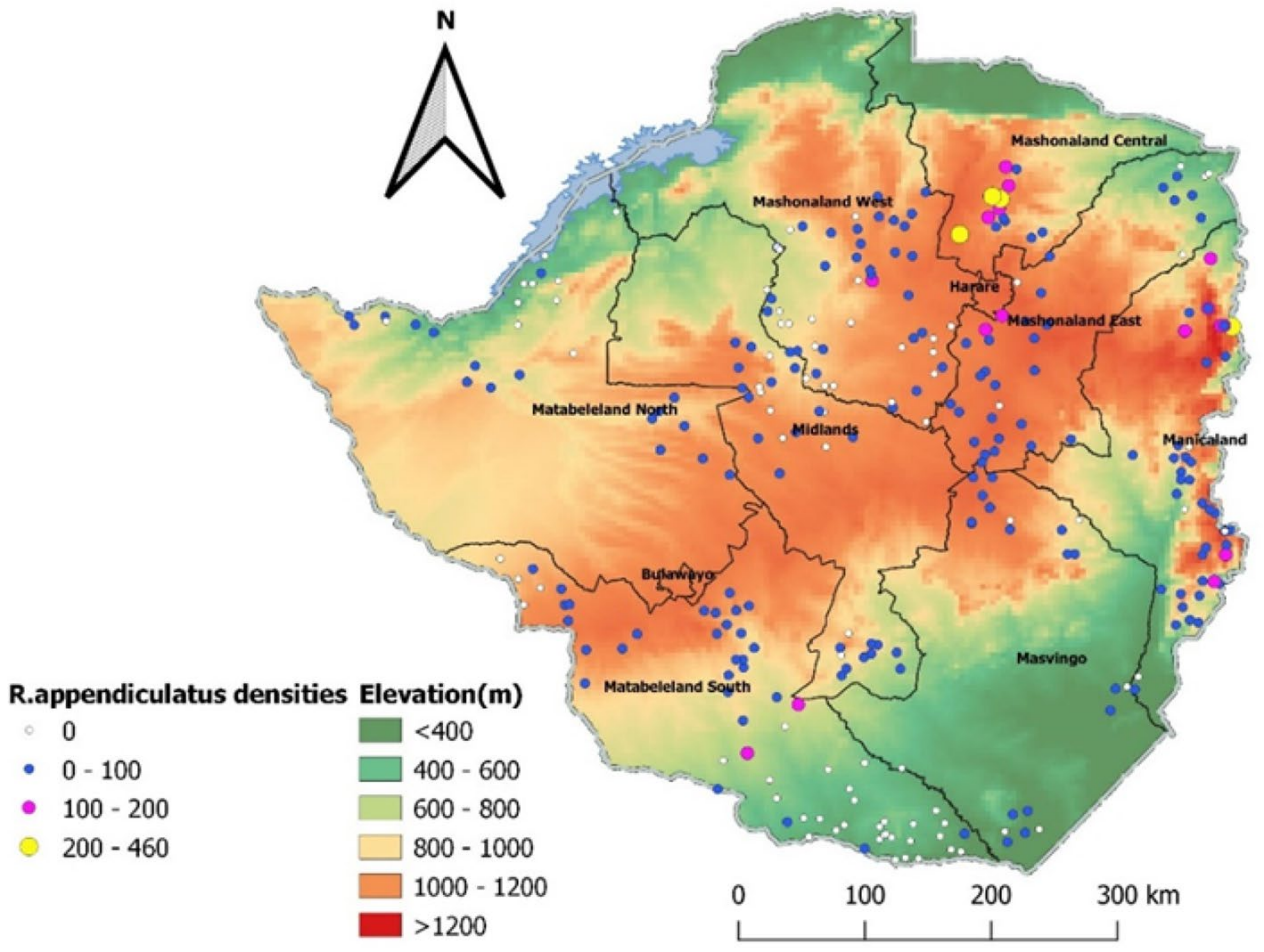
Distribution and presence locations of *Rhipicephalus appendiculatus* in relation to elevation during the 2013 national tick survey

**Fig. 6:**
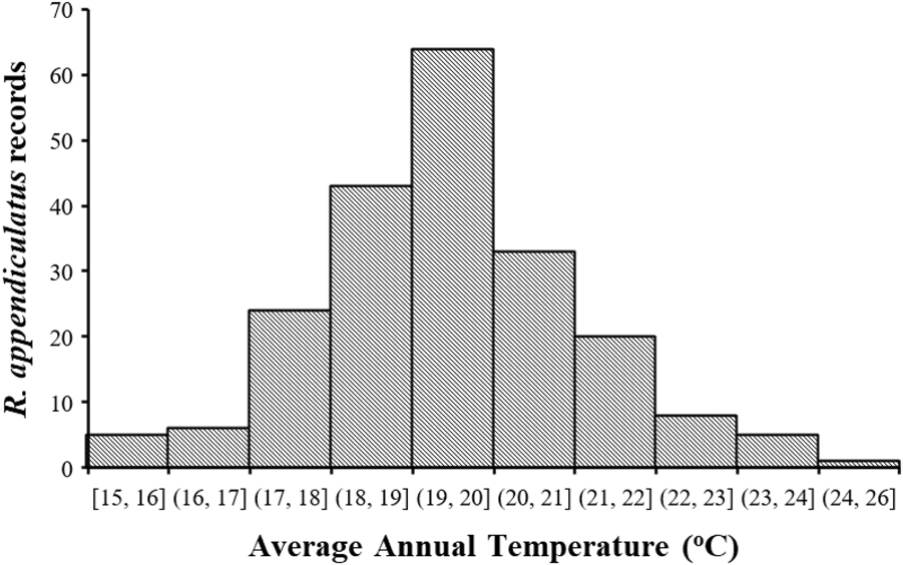
Relationship between occurrence of *Rhipicephalus appendiculatus* and average annual temperature in Zimbabwe

The observed distribution of *R. appendiculatus* based on 2013 presence data is mainly confined to the high elevation regions (> 600 m asl) (Fig. 5). These areas mainly constitute the interior regions although some presence records were also recorded within the marginal regions of Zimbabwe. Although the tick was found across all ecological regions, it was rarely found in low lying areas (< 400 m asl) and distribution also decreased towards the extreme southern and western parts of the country characterized by hot-dry weather conditions. Highest densities of the tick were found in Mashonaland Central to the northern side of the country (Fig. 5). Most of the *R. appendiculatus* occurrence locations were associated with temperatures between 15 and 26 °C with the highest frequency of occurrence records coinciding with the 19–20 °C range (Fig. 6).

### Current and future distribution of suitable habitats for ***Rhipicephalus appendiculatus***

**Fig. 7:**
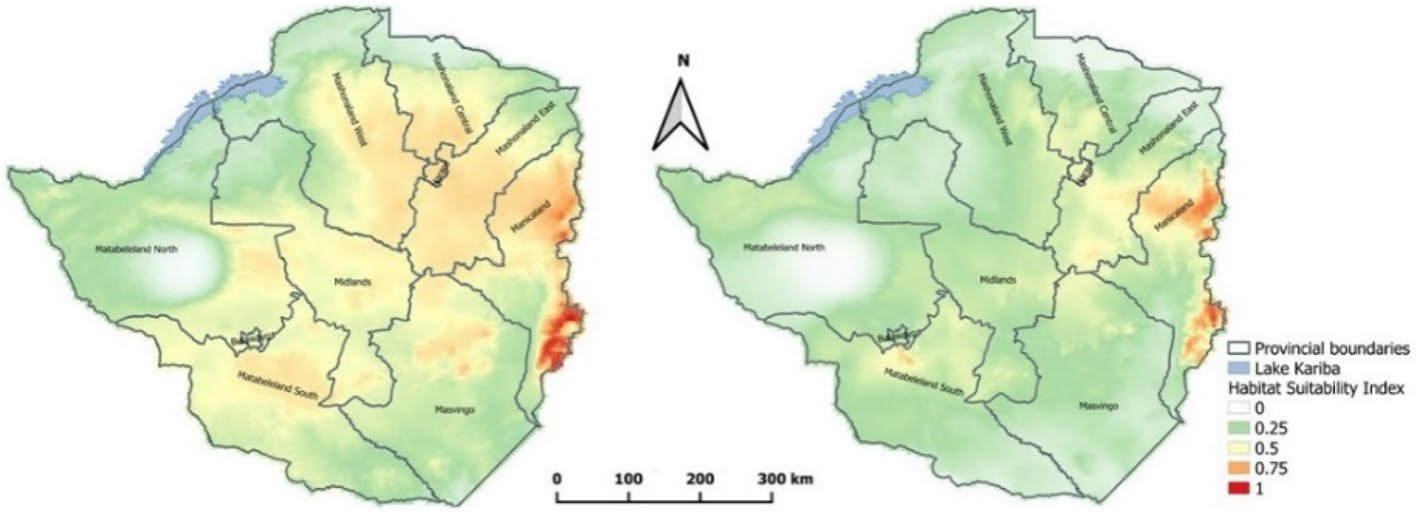
Suitable habitats for *Rhipicephalus appendiculatus* under current environmental conditions (**a**) and the Shared Socioeconomic Pathway 2 (SSP2-4.5) 2041–2060 climate scenario (**b**)

The potential distribution of *R. appendiculatus* based on habitat suitability indices under prevailing climatic conditions indicate that the suitable range of the tick is very wide despite spatial differences in suitability indexes. The eastern parts of the country, which experience low average annual temperatures, had the highest habitat suitability indices, followed by southeastern and central parts of the country including Masvingo and Mashonaland provinces. (Fig. 7a). The extreme southern and northern parts of the country which are characterized by high average annual temperatures had the least suitable habitats despite having isolated records of *R. appendiculatus* presence. However, a significant reduction in areas suitable for *R. appendiculatus* is expected by 2050 if temperatures rise by 2.7 °C as predicted by the SSP2-4.5 global circulation model. Areas with highest suitability indices will be limited to the eastern highlands whereas Mashonaland East, Midlands and parts of Matebeleland South province will become moderately suitable (Fig. 7b). The AUC for both training and evaluation was 0.66 whereas the continuous Boyce Index was 0.96.

## Discussion

This study predicted a wide geographic range for *R. appendiculatus* in Zimbabwe under current climatic conditions. However, this distribution is expected to shrink with a 2.7 °C rise in temperature by 2050 as predicted by the SSP2-4.5. These results indicate that despite the widespread distribution of the tick observed currently, this is constrained by climatic conditions. Previously, the distribution of ticks in Zimbabwe was influenced by land-use practices with *R. appendiculatus* commonly occurring in commercial farms (Norval 1979). However, this study has shown additional areas where the tick is likely to establish in view of changes in land tenure effected in Zimbabwe. These predicted suitable habitats are mainly based on climatic conditions. Several authors have also noted the importance of climate on tick survival, development, behavior, activity and pathogen incubation and transmission (Hunter 2003; Dantas-Torres and Otranto 2011; Bellard et al. 2012). In this regard, the model gives an insight into areas which may require attention during surveys for *R. apendiculatus* and responses to theileriosis outbreaks. Moreover, climatic conditions are expected to play a critical role in moderating future tick distributions as Sungirai et al. (2018) also predicted a reduction in suitable habitats for a congeneric tick, *R. microplus* in Zimbabwe. Danielova et al. (2010) suggested that rising temperatures may expand both the altitudinal and latitudinal ranges of tick species as they observed the occurrence of *Ixodes ricinus* shift, from 750 to > 1000 m asl when temperatures rose by 1.4 ºC over 2 decades. However, in our case *R. appendiculatus* already occupies the high-altitude areas which are characterized by lower temperatures and it is expected to remain in those areas in the future.

This study further revealed the utility of modelling in understanding current species distributions for operational purposes. Sampling particularly at wider geographical scales is often logistically challenging hence modelling can be used to infer the distribution of a particular organism from presence locations. This is particularly important in this study as questions have been arising on the factors modulating the occurrence of theileriosis, a bovine disease transmitted by *R. appendiculatus.* Therefore, the habitat suitability model helps explain these disease incidences as it shows that the tick can survive in most Highveld areas of Zimbabwe. As the dataset used in modelling suitable habitats for *R. appendiculatus* was collected a decade ago, this model explains the occurrence of the diseases transmitted by the tick in areas previously classified as non-endemic. Species distribution models have been previously used to optimize large-scale vector control programs (Dicko et al. 2014) where species-specific interventions can be strengthened. However, many countries lack long-term replicated data on tick abundance, distribution and prevalence (Nuttall 2021).

Results of thermal assays using static protocols, showed that temperatures (ULT_50_) above 44 °C are lethal for *R. appendiculatus* larvae. Zimbabwe has in recent years been experiencing flushes of high temperatures (heat waves) with a record high of 46.5 °C in Chipinge District, South-East Zimbabwe, in 2015. Ticks as ectotherms are expected to be affected by these extreme temperatures. However, the lethal temperatures recorded in this study were well above the microhabitat temperatures measured in selected districts, further indicating that the tick species can survive current climatic conditions. This has implications on diseases transmission dynamics as the success of disease vectors such as ticks is often determined by the ability to survive in unfavorable conditions such as temperature fluctuations that directly affect their physiology and behavior (Gilbert et al. 2014; Rosendale et al. 2016). Although thermal tolerance, physiological mechanisms and thresholds in relation to survival may vary from one developmental stage to another (Holmes et al. 2018; Mutamiswa et al. 2019), this study showed that there is little variation in lethal temperature between larvae, nymphs and adults. However, the larval stage is the most vulnerable as it may be exposed to extreme temperatures on the host as opposed to the other stages which may seek refuge under plant litter. Questing larvae, nymphs or adults have been observed to withdraw into refugia as a survival mechanism against high temperatures (Hove et al. 2008).

In conclusion, this study has shown that *R. appendiculatus* can survive under a wide range of climatic conditions. However, its distribution is expected to be restricted in future with an increase in temperatures as indicated by reduction in areas with suitable habitats. Therefore, under current climatic conditions, it should be expected that the tick should disperse and establish into these habitats thus increasing the probability of occurrence of the associated tick-borne diseases. These findings justify the need to review the national tick control strategies considering the predicted current wide distribution.

## Data Availability

All relevant data is provided within the manuscript.
